# Dimeric architecture and membrane thinning govern substrate recognition by human signal peptide peptidase

**DOI:** 10.21203/rs.3.rs-9372626/v1

**Published:** 2026-06-01

**Authors:** Owain J. Bryant, Nikita Sergejevs, Fiorin Giacomo, Jacob Robson-Tull, Justin C. Deme, Lucy R. Forrest, Pedro Carvalho, Susan M. Lea

**Affiliations:** 1 Structural Biology, St Jude Children’s Research Hospital, Memphis, TN 38105, USA.; 2 Center for Structural Biology, Center for Cancer Research, National Cancer Institute, Frederick, MD 21702, USA.; 3 Sir William Dunn School of Pathology, University of Oxford, Oxford, UK.; 4 Computational Structural Biology Section, National Institute of Neurological Disorders and Stroke, National Institutes of Health, Bethesda, MD 20892.

## Abstract

Signal peptide peptidase (SPP) is an intramembrane aspartyl protease that cleaves transmembrane segments of diverse origin, such as remnant signal peptides of secretory proteins or transmembrane helices of tail-anchored proteins. Consistent with its ability to cleave multiple substrates, SPP is essential in mammals, and its activity is linked to a wide range of processes from immune regulation to protein quality control and cancer. Here we determine cryo-EM structures of human SPP in its apo form and bound to a signal peptide substrate. Together with molecular dynamics simulations and functional assays, our data show that SPP forms a constitutive homodimer that locally curves and thins the membrane, placing the conserved active site within the bilayer. Substrate engagement drives major conformational changes, including movement of a “latch” helix that positions the signal peptide for cleavage. The substrate adopts a tilted helix–unwound–β-strand architecture that defines the cleavage register independently of sequence. Steric exclusion by folded luminal domains or additional transmembrane helices explains selective processing of certain protein termini.

Integral membrane and secretory proteins represent more than one-third of the eukaryotic proteome and have their biogenesis at the endoplasmic reticulum (ER)^1–3^. Their targeting and insertion into the ER is mediated by N-terminal signal peptides that form transmembrane (TM) helices during biogenesis^4–8^. Following insertion of the nascent polypeptide, signal peptidase (SPase) usually cleaves off the signal sequence from the precursor protein, leaving a remnant signal peptide TM helix embedded in the membrane with a type-II topology (Ncyt and Clum)^8–11^ (Figure 1A). This remnant signal peptide is subsequently processed by signal peptide peptidase (SPP), an intramembrane aspartyl protease that hydrolyses the peptide backbone within the lipid bilayer releasing two small fragments^11–14^ (Figure 1A). Although the fate of these fragments is not fully understood, some are loaded onto MHC-I complexes for antigen presentation at the cell surface, highlighting an important role of SPP in immune regulation^11,13^.

Beyond signal peptides, SPP cleaves a variety of other type-II TM segments as part of cellular quality control processes such as ER-associated protein degradation (ERAD)^13–19^ and, in infected cells, some viral core proteins (including hepatitis C and human cytomegalovirus)^18,20^. SPP is essential in mammals, with genetic deletion in mice resulting in embryonic lethality and its dysregulation is linked to altered immune signalling, viral pathogenesis and elevated expression in many human breast and lung cancers, highlighting its clinical importance^21–24^.

SPP belongs to the GxGD-type family of intramembrane proteases, which includes the presenilin’s – the catalytic subunit of the γ-secretase complex that is crucial in processing substrates such as the Notch receptor (linked to several cancers) and amyloid precursor protein (central in the pathogenesis of Alzheimer’s disease)^13,25–27^. Both SPP and γ-secretase contain an active site and perform proteolysis within the hydrophobic environment of the membrane^13,14,28,29^. Despite mechanistic similarities, γ-secretase activity is tightly regulated through multi-subunit assembly, presenilin autoproteolysis and trafficking to the correct cellular compartments, whereas SPP is catalytically active on its own, indicating that SPP activity is regulated by alternative mechanisms^30–35^. However, the architecture of SPP and the mechanism by which it achieves selective substrate recognition are not understood^36,37^. Here we address these key questions by determining cryo-EM structures of SPP, both in isolation and with bound substrate, combined with molecular dynamic simulations and cell-based functional assays.

## Structure of human signal peptide peptidase

**Figure 1:**
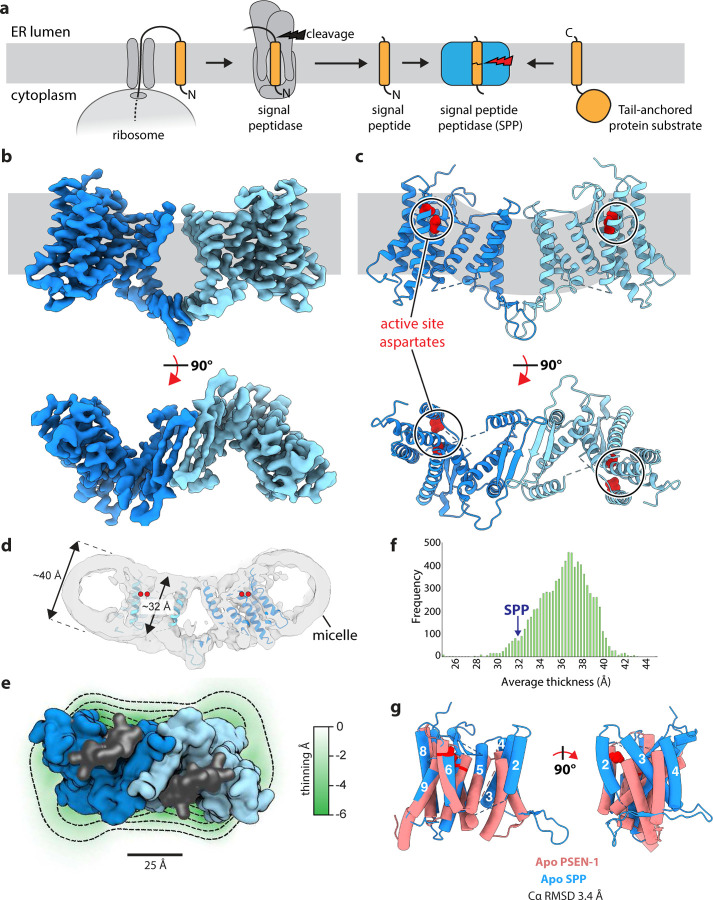
Architecture of human Signal Peptide Peptidase. **a**, Nascent secretory and membrane proteins often possess N-terminal signal peptides that mediate targeting and co-translational insertion into or transport across the ER membrane. Following insertion, the signal peptide is removed by the signal peptidase complex, releasing the mature protein. Signal Peptide Peptidase (SPP) subsequently cleaves the remaining signal peptide within the ER membrane. In addition to signal peptides, SPP also cleaves other transmembrane helices at protein termini, such as in tail-anchored proteins. **b**, Cryo-EM volume of human SPP in DDM/CHS at 3.8 Å resolution (contour level 0.195), viewed from the membrane plane (top) or from the cytoplasm (bottom). Individual protomers are indicated in light and dark blue. **c**, Cartoon models of human SPP viewed from the plane of the membrane (top) or from the cytoplasm (bottom). Catalytic aspartate residues (D219 and D265) are coloured in red and depicted as spheres. **d**, Cartoon model of SPP docked into the cryo-EM volume of SPP. The detergent micelle (grey; contour level 0.05) indicates the position of the membrane. The location of the catalytic aspartate residues are depicted as red spheres. **e**, Membrane deformation maps from coarse-grained MD simulations of the bilayer midplane around the apo SPP dimer. Lateral coordinates are in Å units; grid spacing approximately 3 Å. Protein density is show in blue (experimentally determined) and grey (loops absent in experimental structure). **f**, The Distribution of average membrane thickness values for the annular shell (within 7 Å) around the membrane proteins in the MemProtMD database. The narrowest thickness for SPP is highlighted. **g**, Structural alignment of the SPP model presented in this study (blue) with Prenesilin-1 (red; PDB 5A63). Residues 182–333 of SPP were used for alignment. Alignments and RMSD were calculated using the MatchMaker command in ChimeraX. Catalytic aspartate residues are coloured in red and depicted as spheres

Following extensive detergent screening and sample optimisation, we determined the apo structure of SPP using single particle cryo-EM to an overall resolution of 3.8 Å, with the core resolved to ~3.2 Å resolution (Figures 1B–1C, S1A-S2B Table S1). Initial 2D class averages showed that particles displayed preferential orientations; to improve this we combined additional datasets with tilting, sample supplementation with fluorinated octyl maltoside or PEGylated protein immediately prior to grid preparation (Figure S1). The final structure revealed a dimeric organisation, stabilised by two key interfaces: a β-sheet on the cytoplasmic side of the ER membrane and a Phe96-Leu178 contact on the luminal side (Figures 1B–1C, S2A-B). This β-sheet stabilises dimer formation, and its deletion results in lower SPP levels (figure S2C). The oligomeric state of SPP was previously unclear, with a range of states proposed in the literature^36,37^. However, we were unable to detect any particles corresponding to assemblies larger or smaller than a dimer (Fig. S1). Each copy of SPP consists of nine TM helices, two β-strands located at the cytoplasmic side of the membrane and two β-strands located in the plane of the membrane (β1 and β2) proximal to the pair of conserved catalytic aspartate residues (Asp219 and Asp265) (Fig. 1C, S3A). Only 178 residues of each SPP copy are ordered, corresponding to an ordered mass of ~39 kDa, which helps explain why structure determination was so challenging (Fig. S1). The TMs are physically separated at the cytoplasmic side of the membrane by the inter-subunit β-sheet, but make contacts at the luminal side, tilting the subunits by an angle of 22° degrees relative to the membrane normal (and 44° relative to each other) creating a lipid filled triangular opening between the two subunits (Figures 1B–C, S2A, 3A). This tilt generates substantial membrane curvature, evident from the shape of the detergent micelle and also seen in molecular dynamics (MD) simulations of the dimer starting from a planar model bilayer (Figures 1D–E, S3). TMs 2–5, which lie closest to the dimer interface, show the largest tilt, resulting in local membrane thinning to ~32 Å width compared to the typical ~40 Å width of the ER membrane (Figures 1D, 1E). Structural alignment of SPP with the related presenilin component of the γ-secretase complex shows that SPP TM1 and TMs 5–9 align with presenilin with an overall RMSD of 3.4 Å for CA pairs, whilst TMs 2–4 do not align with TMs in presenilin (Figures 1G, S3D-S3E). Most notably, the position of the catalytic aspartates and other signature motifs of these proteases, such as ΦD, GxGD and PAL, are well conserved between the structures^38,39^. Despite these similarities, SPP and presenilin adopt opposite membrane topologies with the catalytic residues proximal to the luminal surface of the ER membrane in SPP while in presenilin they are within the cytosolic membrane leaflet (Figures S3D-S3E). These observations suggest that a conserved intramembrane protease fold has evolved to adopt opposite membrane topologies, enabling substrate processing from either leaflet of the bilayer.

The most extensive surface involved in SPP dimerization is a β-sheet on the cytoplasmic side of the membrane formed from a 33-residue insertion between TM3 and TM4 (Figures S4). While other members of the SPPL family contain insertions at a similar position, these are shorter.

SPPL2A is experimentally shown to be monomeric in DDM detergent and the other members of the family do not form beta-sheet mediated dimers in alphafold predictions^40^. Thus, dimerization induced by this interface appears to be unique to SPP among all family members.

## Structural basis for signal peptide recognition and processing by SPP

**Figure 2:**
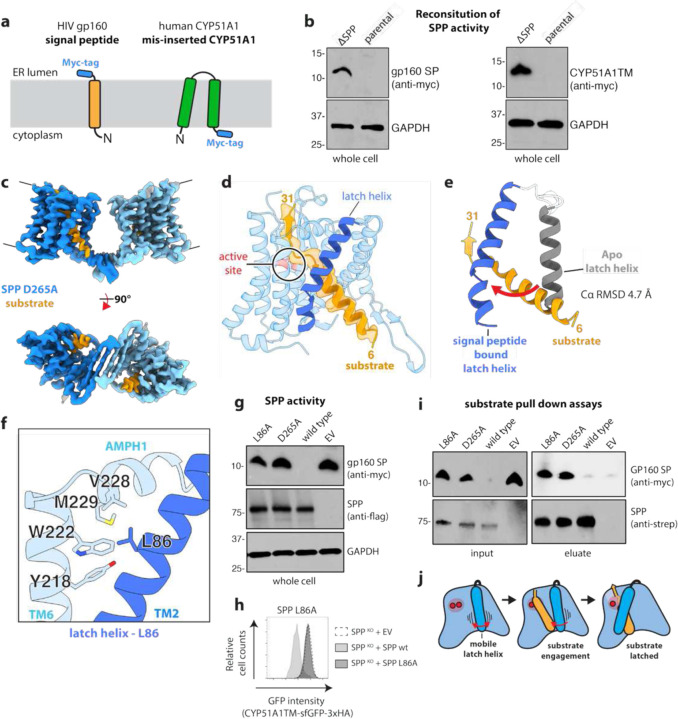
Structural basis of signal peptide recognition by human SPP. **a**, Schematic representations of two SPP substrates: The signal peptide of Human Immunodeficiency Virus (HIV) gp160 (orange; left) and the N-terminal metastable helix of human lanosterol demethylase (CYP51A1; green; right). C-terminal Myc-tags were fused to each polypeptides and used for immunodetection in downstream assays. **b**, SPP proteolytic activity was assessed in HEK293 cells. SPP knock out (ΔSPP) and Parental control (Parental) cells were transfected with vectors encoding either the signal peptide of HIV gp160 (left) or the N-terminal fragment of human CYP51A1 (right) and incubated for 48 hours. Whole cell lysates were analysed by SDS-PAGE followed by immunoblotting. GAPDH was used as a loading control. **c**, Cryo-EM volume of human SPP (blue) in complex with the signal peptide of HIV gp160 (orange) at 4.0 Å resolution (contour level 0.23), viewed from the membrane plane (top) or from the cytoplasm (bottom). **d**, One SPP-signal peptide unit viewed from the plane of the membrane. The HIV gp160 signal peptide is shown in the cryo-EM volume (transparent orange surface). The latch helix (TM2) is colored dark blue. The location of the catalytic aspartate residues are highlighted in red. **e**, Structural alignment of the apo- (grey) and signal peptide- bound SPP complexes (blue and orange) highlighting the large conformation change in the latch helix (arrow shows movement of the latch helix upon signal peptide binding). Alignments and RMSD were calculated using the MatchMaker command in ChimeraX. **f**, Leucine at position 86 (L86) within the SPP latch helix (TMH2) makes multiple contacts with residues in SPP TM6 and AMPH1 when in the substrate bound conformation. **g**, Reconstitution of SPP activity in HEK293 SPP Knock out cells (top). Cells were co-transfected with plasmids encoding the signal peptide of HIV gp160 and an empty vector, flag tagged SPP wild type or its variants (L86A, D265A). Whole cell lysates were analysed by SDS-PAGE followed by immunoblotting. GAPDH was used as a loading control. **h**, Flow cytometry analysis of substrate degradation in SPP KO cells expressing the CYP51A1TM–GFP model substrate from a doxycycline-inducible locus (bottom). Besides the fluorescent model substrate, SPP KO cells were transduced with an empty vector (dotted line) or plasmids encoding SPP wild-type (WT, light grey) or L86A (dark grey). **i**, Analysis of HIV gp160 signal peptide binding to SPP wild type, catalytically inactive (D265A) and L86A mutant. Binding was assessed by the amount of gp160 pulled down by resin-immobilised SPP. Proteins were detected by immunoblotting. SPP(D265A) was used as a positive control and wild type SPP (which cleaves bound signal peptides) was used as a negative control. **j**, Schematic showing the movement of the SPP latch helix upon signal peptide binding.

To study substrate processing by SPP, we developed a cell-based substrate cleavage assay using simplified tagged substrates to allow detection of enzyme activity by immunoblotting. We chose two model substrates: the signal peptide of human immunodeficiency virus (HIV) gp160 (a precursor glycoprotein for the viral envelope)^41^ and residues 1–61 of human CYP51A1 (lanosterol demethylase), whose cleavage by SPP triggers CYP51A1 quality control^42^ (Figure 2A). This CYP51A1 sequence contains two predicted helices but these are predicted to interact independently with the membrane rather than packed together, and it is the N-terminal helix that is the target for SPP. These peptides were designed to directly monitor SPP activity without requiring pre-processing by SPase and engineered to carry a C-terminal Myc tag to monitor their intracellular levels prior to cleavage. Upon cleavage, the tag becomes undetectable, presumably due to rapid clearance of the short fragment generated by SPP cleavage. Both model substrates accumulate in SPP-deficient HEK293 cells but are absent in control cells, consistent with SPP-dependent cleavage and clearance, demonstrating that these peptides are recognised and processed by SPP (Figure 2B).

To obtain structural insight into signal peptide recognition by SPP, we attempted to purify SPP-substrate complexes from cells co-expressing SPP with either the HIV gp160 signal peptide or human CYP51A1TM residues 1–61. To increase the stability of the SPP-substrate complex, the catalytic Asp265 was mutated to alanine, rendering SPP proteolytically inactive. In this background a SPP-substrate complex could be purified although substrate gradually dissociated during purification, therefore membrane solubilisation, tandem-affinity purification, gel filtration and grid preparation were performed in a single, uninterrupted, workflow, limiting the amount of dissociated complex in the final sample. The SPP-CYP51A1TM complex dissociated during the dilution caused by the final size-exclusion chromatography step, whereas the SPP-gp160 complex remained intact and could be analysed by cryo-EM, yielding a final volume at an overall resolution of 4.0 Å, with the core resolved to ~3.2 Å resolution (Figures 2C, S5, Table S1). Densities corresponding to two copies of the gp160 signal peptide are well resolved in the dimeric SPP complex, with visible density corresponding to residues 6–31, allowing confident model building (Figures 2C–2D, S5, Table S1). The signal peptide runs from the SPP β-sheet located at the dimer interface towards the periphery of the complex at a 45° angle relative to the membrane plane (Fig. 2D). Like the apo SPP structure, the substrate bound SPP complex shows substantial membrane thinning and curvature, evident from the shape of the detergent micelle and also seen in MD simulations of the dimer starting from a planar model bilayer (Figures S5-S6). The substrate bound structure reveals that SPP undergoes dramatic conformational changes (Cα RMSD 4.7 Å) and multiple regions not visible in the apo map become ordered (Figures 2E, S7A). The most striking conformational change occurs in TM2 (hereafter^38^erred to as the ‘latch helix’), where it moves within the membrane by 19.5 Å to clamp the signal peptide in place (Figures 2E, 7A). In addition to the large conformational change, the latch helix displays substantial mobility in the substrate-free state, which is substantially reduced in the substrate-latched state, as reflected by a decrease in B-factors upon substrate binding (Figure S7B) and additional residues (residues 67–78) become ordered upon substrate binding (Figure S7A). Other conformational changes include movements of TMs 6 and 8 at the luminal side of the membrane towards the signal peptide, and residues 228–235 become ordered to form an amphipathic helix at the luminal side of the membrane (AMPH1, Figure 2F, S7A).

To assess the functional importance of the latch, we substituted Leu86 - positioned within the latch helix and contacting SPP TM6 and AMPH1 signal peptide bound/latched state - with alanine. This SPP L86A mutant was inactive in cleaving gp160 based on our cell-based assay (Figure 2G). The SPP L86A mutant was also deficient in triggering degradation of CYP51A1TM–sfGFP–3xHA, a quality control substrate targeted by ERAD upon SPP-cleavage 42 (Figure 2H). Importantly, L86A mutation does not impair SPP ability to bind substrates (Fig. 2I, S7C), indicating that the latch helix is not required for initial docking but is essential to trap substrates in a proteolysis-competent position (Figure 2J).

## Defining the structural requirements of the SPP active site.

**Figure 3:**
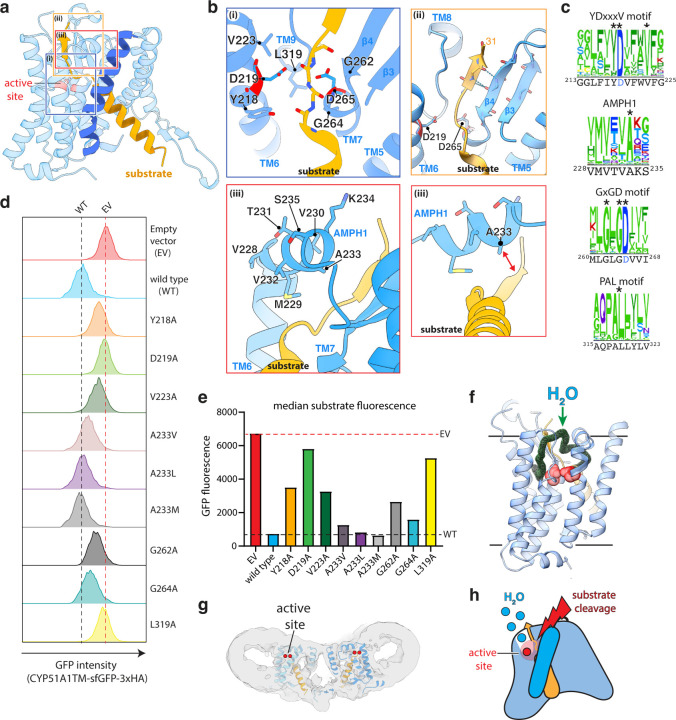
Structural determinants of signal peptide cleavage by SPP. **a**, Cartoon representation of a single unit of the SPP-signal peptide complex as in figure 2D. Boxes indicated regions enlarged in b. **b**, Zoomed in view of the active site (i, top left), the hybrid beta sheet formed between substrate and SPP beta strands 3 and 4 (ii, top right) and of SPP AMPH1 that becomes ordered upon signal peptide highlighting the highly conserved A233 and its proximity to the signal peptide. (iii, bottom left to right). SPP is coloured in blue, signal peptide in orange. **c**, Sequence logos of conserved SPP motifs including YDxxxV in TM6, AMPH1 highlighting the highly conserved A233, the GxGD in TM7 and the PAL in TM9. Amino acids are coloured according to their physicochemical properties: hydrophobic and neutral (green), polar (light blue), positive charge (dark blue) or negative charge (purple). **d**, Flow cytometry analysis of substrate degradation in SPP KO cells expressing the CYP51A1TM–GFP model substrate from a doxycycline-inducible locus (bottom). Besides the fluorescent model substrate, SPP KO cells were transduced with an empty vector or plasmids encoding SPP wild-type or the indicated SPP mutant. **e**, Bar chart showing the median CYP51A1TM–GFP fluorescence intensities from Figure 4D. **f**, Cartoon model of a single SPP subunit indicating the water channels identified with CAVER and visualised as a mesh (green). The location of the catalytic aspartate residues are depicted as spheres. **g**, Cartoon model of SPP docked into the cryo-EM volume of SPP viewed from the plane of the membrane. The detergent micelle (grey; contour level 0.05) indicates the position of the membrane. The location of the catalytic aspartate residues are depicted as spheres. **h**, Schematic showing the water accessible hydrophilic cavity that leads to the active site and that enables SPP proteolytic activity.

The substrate bound SPP structure shows that the bound signal peptide comprises three structural elements: a TM helix (residues 8–24), a short-unwound segment (residues 25–27) and a C-terminal β-strand (residues 28–30) (Figures 3A–B, S7A). The TM helix is enclosed by SPP helices 2, 3, 5, 7 together with AMPH1 (Figures 3A–B, S7A), whilst the unwound segment immediately following the helix sits directly at the active site, exposing the normally buried scissile peptide bond for catalysis (Fig. 3B). The C-terminal β-strand of the substrate pairs with SPP β3 and β4 strands to form a hybrid β-sheet on the luminal side of the membrane, anchoring the C-terminal portion of the signal peptide in place (Figure 3B) and thereby defining substrate positioning relative to the active site. Two previously identified SPP amino acid sequence motifs (YD and GxGD) that contain the catalytic aspartates (D219 and D265), are positioned in TM6 and TM7 and sandwich the unwound segment of the signal peptide^11^ (Fig. 3B, 3C). A third sequence motif, the PAL motif, previously thought to be positioned away from the active site, is instead positioned adjacent to the YD and GxGD motifs and may stabilise them^11^ (Figure 3Bi). Using the same flow cytometry-based assay, we showed that other SPP mutants had a range of processing defects, from no detectable defect (A233L, A233M) to mild (G264A, A233V), strong (Y218A, V223A, G262A) or severe (D219A and L319A) loss of activity. Importantly, these phenotypes are not due to impaired expression as all mutants were present at levels comparable to wild type SPP (SXXX). Substrate binding results in the ordering of residues 228–235 at the luminal side of the membrane into an amphipathic helix (AMPH1) (Figures 3C, 3F). Notably, sequence alignments revealed a highly conserved residue, A233, positioned proximal to the unwound portion of the signal peptide (Figures 3B–C). We wondered if a small residue at this position would prevent a clash with substrates (Figure 3B). Despite the high level of conservation, mutation of A233 to Leucine or methionine had no effect on substrate processing, whilst mutation to Valine had a mild substrate processing defect (Figure 3D–E) suggesting that this helical element can rearrange to accommodate modest side chain changes.

Structural alignment of our SPP-substrate complex with the related presenilin component of γ-secretase bound to either Notch (Fig. S7E) or APP (Figure S7F) demonstrates clear parallels in the modes of substrate engagement, with γ-secretase substrates adopting a similar extended conformation (Figure S7A). Unlike in SPP, the region equivalent to the latch helix in presenilin is fully disordered in the unbound state of γ-secretase^43,44^.

**Figure 4:**
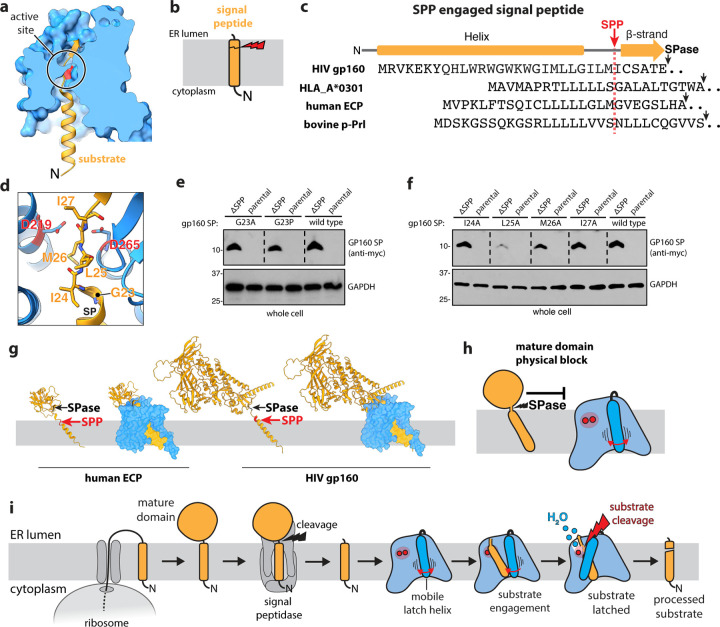
Substrate processing shows limited sequence dependence and is constrained by steric features **a**, Slice through view of the surface model of SPP (blue) and cartoon model of the signal peptide (orange) for one SPP-signal peptide unit. The catalytic aspartate residues are coloured red. **b**, Schematic of a typical signal peptide with the site of SPP-mediated cleavage indicated. **c**, Schematic representation of HIV gp160 signal peptide with secondary structural elements derived from the SPP bound structure (top). Sequences of four validated SPP signal peptide substrates aligned to the SPP cleavage site are also shown (bottom, red arrow and dotted line). The signal peptidase cleavage site is indicated with a black arrow. **d**, Zoomed in view of the HIV gp160 signal peptide (orange) at the active site of SPP (blue), highlighting signal peptide residues surrounding the catalytic aspartate residues (red) of SPP. **e and f**, SPP proteolytic activity was assessed in HEK293 cells. SPP knock out (ΔSPP) and Parental control (Parental) cells were transfected with vectors encoding wild type signal peptide of HIV gp160 or the indicated variants and incubated for 48 hours. Whole cell lysates were analysed by SDS-PAGE followed by immunoblotting. GAPDH was used as a loading control. **g**, Structural alignment of the AlphaFold predicted structure of full-length human Eosinophil Cationic Protein (ECP, left) or the AlphaFold predicted structure of full-length HIV gp160 (right) on our cryo-EM structure of the signal peptide bound SPP complex. In both cases, the folded luminal domains of the full-length substrates sterically clash with SPP, providing an explanation for why only signal peptide remnants are recognised and processed by SPP. **h**, Schematic showing the steric clash between the folded luminal domain of a pre-protein and SPP. **i**, Updated model for the mechanism of signal peptide processing by SPP based on the results of this study. Nascent secretory and membrane proteins that possess N-terminal signal peptides are co-translationally inserted into or transported across the ER membrane. Following insertion, signal peptidase cleaves the signal peptide from the precursor protein, releasing the mature protein. Only after removal of the mature protein is the remnant signal peptide recognized by SPP. Signal peptide binding induces large conformational changes in SPP including the movement of a latch helix that locks the signal peptide in place. Conformational changes in SPP due to substrate engagement also generate a hydrophilic channel that allows water molecules to access the active site for proteolytic cleavage. Upon cleavage the latch helix becomes mobile allowing release of the proteolytic fragments.

How water molecules, essential for catalysis, access the SPP active site within the hydrophobic bilayer is unclear^11,14,28^ (Figures 3F–G). Despite visible membrane thinning in the SPP apo volume (Fig. 1D), the micelle does not thin sufficiently to expose the active site. Intriguingly, ordering of AMPH1 associated with binding of substrate introduces an additional hydrophilic cavity in the detergent micelle leading to the active site that can accommodate waters providing access to the active site (Figures 3F, 4A–B, Fig S8).

Comparative analyses show that signal peptides are highly variability in sequence^45,46^ (Figure 4C). Nevertheless, a common feature of SPP substrates is the presence of helix breaking residues in the TM region such as proline or glycine residues, although their position relative to the scissile bond is not absolutely conserved^14^. The HIV gp160 signal peptide contains such a residue (G23), at the C-terminal end of the TM helix, immediately preceding the unstructured region (Fig. 4D). Substituting G23 with either the highly helix-breaking proline or helix-favouring alanine did not impair SPP-dependent processing (Figure 4E). This supports the idea that the only requirement is exposure of the scissile bond for attack rather than unwinding of the TM helix from a specific point with the register of the substrate within the active site driven by formation of the β-sheet C-terminal to the scissile bond. Our structure reveals that residues in the unwound region (L25-M26-I27) pack tightly between TMHs 6 and 7 and AMPH1, aiding position of the backbone for cleavage (Figure 4D). To test their functional importance, these residues and the immediately flanking positions were mutated to alanine and analysed for SPP-mediated processing using our cell-based substrate cleavage assay. Although the L25A mutant accumulated to lower levels in SPP-deficient cells, suggesting reduced intrinsic stability, none of the mutants were detectable in wild type cells, indicating that all were efficiently cleaved by SPP (Figure 4F). We next examined how altering signal peptide length affects processing by SPP. Deletion of four or eight residues upstream the cleavage site (positions 16–19 or 12–19) reduced signal peptide accumulation in SPP-deficient cells-suggesting reduced intrinsic stability, whereas neither mutant was detectable in wild type cells (Fig S9A-E). Similarly, inserting four or eight residues before the cleavage site (ALIL or ALILALIL) between positions 19 and 20 produced variants that were also undetectable in wild type cells, indicating that all these signal peptides remained competent for SPP processing (Figures S9D-E). Together, these results show that SPP can efficiently process a wide variety of sequences tolerating extensive changes in both sequence composition and length upstream the cleavage site. Consistent with this, analysis of the B-factors of the signal peptide bound to SPP reveals that the helical region preceding the cleavage site exhibits higher B-factors – and thus greater mobility – compared with the β-strand that anchors the signal peptide downstream of the cleavage site (Figure S9F).

## Mature domains of pre-proteins prevent signal peptide binding to SPP

The ability of SPP to process a wide range of sequence divergent substrates highlights the major outstanding question - how does SPP achieve selectivity for signal peptides only after the removal of the mature domain by signal peptidase? A similar principle operates in γ-secretase, which recognises substrates only after prior cleavage by other enzymes, with the nicastrin subunit acting as a major discriminator by specifically recognising substrates with an appropriately short extracellular domain^34^ (generated by a preceding proteolytic step^47^). Similarly, during preparation of this manuscript, a structure for SPPL2a was published revealing that other members of the SPP family are monomers with folded luminal domains that likely also contribute to substrate specificity^40^. Unlike γ-secretase, SPP is active without any other binding partners, and unlike SPPL2a, it lacks other extramembrane domains that could function analogously to nicastrin^33,40^. Our structures provide a simple and elegant explanation for why SPP selectively recognises and cleaves only remnant signal peptides or helices at protein termini. Signal peptidase (SPase) cleaves signal peptides on the luminal side of the ER membrane-very close to the membrane surface (with the active site positioned only ~10–15 Å away) -leaving only a short segment in the ER lumen^48^. Structural alignments of AlphaFold-predicted structures (e.g. human ECP or HIV gp160) reveals that the folded mature domains sterically clash with SPP, preventing signal peptide binding unless the signal peptide has been released from the folded domain (Figures 4G–H). This steric mechanism provides a means of distinguishing remnant signal peptides from intact pre-proteins by SPP. Tail-anchored proteins lack a folded C-terminal luminal domain that could clash with SPP, explaining how many tail-anchored proteins can be SPP substrates. Specificity for single-pass TM helices is further achieved by the dimer sterically excluding of TM bundles from multispanning membrane proteins from the active site. Thus, steric exclusion of either the folded mature domain or multispanning membrane proteins provides a mechanism for achieving specificity that does not depend on high levels of sequence conservation in the TM targeted for cleavage.

## Discussion

Our structural and functional analyses provide a unifying mechanistic framework for understanding how SPP achieves selective proteolysis within the ER membrane (Fig 4I). The cryo-EM structures reveal that SPP exists as a stable homodimer, resolving longstanding uncertainty regarding its oligomeric state (Figures 1B–D). Dimerization is mediated by conserved cytoplasmic and luminal interfaces and is accompanied by induced curvature of the membrane. The membrane thinning likely facilitates substrate discrimination and may lower the energetic barrier for peptide bond hydrolysis within the hydrophobic bilayer (Figures 1B–D, S2-S4). This membrane remodeling parallels observations for other intramembrane proteases and underscores the importance of the lipid environment in catalysis^49–52^. One defining feature of signal peptides is that their hydrophobic h-region is, on average, shorter than that of canonical TM helices^53^. Recent structural analysis indicated that the Signal Peptidase Complex, while having the active site and promoting catalysis in the ER lumen, also induces membrane thinning and appears to exploit this feature as a specificity determinant to cleave signal peptides from nascent secretory proteins^48^. While SPP protease has its active site in the ER membrane, thinning may be an unifying feature of substrate selection during intramembrane proteolysis. Membrane thinning by SPP is therefore likely to act as an additional determinant in signal peptide selection.

Comparison of apo and substrate-bound structures shows that SPP has highly dynamic regions (Figure 2C–E)). Substrate engagement induces large conformational rearrangements, most notably the movement of TMH2, which acts as a latch to clamp the signal peptide in a proteolytically competent position (Figure 2E). Functional assays demonstrate that this latch is dispensable for initial substrate binding but essential for catalysis, indicating a multistep recognition mechanism in which docking precedes active-site engagement (Figure 2F–H, S7A-B). Ordering of additional elements, including an amphipathic helix at the luminal membrane interface, creates a hydrophilic access pathway that may facilitate water entry to the active site, addressing a central question in intramembrane proteolysis^11,13,14,28,33,37^ (Figures 3F–G, S8A-C).

The bound signal peptide adopts a conserved architectural solution—a tilted TM helix followed by a locally unwound segment and a C-terminal β-strand that pairs with SPP (Figure 3A–B). This hybrid β-sheet interaction fixes the register of the scissile bond, explaining why SPP tolerates extensive sequence and length variation N-terminal to the cleavage site (Figure 4C). Consistent with this model, mutational analyses reveal that exposure and positioning of the backbone, rather than specific side chains, are the primary determinants of cleavage (Figure 4D–F).

Finally, our structures provide a simple explanation for substrate selectivity. Steric clashes between SPP and folded luminal domains, as well as spatial constraints that preclude accommodation of multiple TM helices, ensure that only remnant signal peptides are processed (Figure 4G–H). Together, these findings distinguish SPP regulation from that of γ-secretase and establish general principles by which intramembrane proteases combine membrane remodeling, conformational dynamics and steric exclusion to achieve specificity.

## Materials and methods

### Purification of the wild type SPP

The gene encoding full-length human SPP was codon-optimized for expression in human cells and synthesized as a DNA fragment to include a C-terminal TEV - mVenus - Twin-Strep tag fusion. The gene fragment was inserted into the pHR-CMV-TetO_2_ lentiviral transfer vector. This vector, alongside packaging and envelope plasmids (psPAX2 and pMD2.G), was transfected into HEK293T Lenti X cells to produce lentivirus. The lentivirus was used to transduce HEK293S GnTI^−^ Tet^R^ cells to generate a stable cell line. Cells were cultured in DMEM media (Gibco) supplemented with 1% L-glutamine and 10 % foetal bovine serum (FBS, Gibco) during adherent growth and in FreeStyle 293 Expression medium (Gibco) supplemented with 1% non-essential amino acids and 1% foetal bovine serum (FBS, Gibco) for growth in suspension. Suspension cells were grown at 37 °C, 130 rpm, and 8% CO_2_ and expression were induced with the addition of doxycycline to 10 μg/mL. Cells were harvested two days post induction by centrifugation (1500 rpm, 10 min, 4°C) and stored at −80°C.

Cell pellets were resuspended in buffer A (50 mM sodium phosphate pH 8.0, 150 mM NaCl and 1 mM EDTA) supplemented with DNase I (30 μg/ml) and a cOmplete EDTA-free Protease Inhibitor Cocktail tablet (Sigma) for 30 minutes before passing through an EmulsiFlex C5 homogenizer (Avestin) at 15,000 psi. Lysates were clarified by centrifugation (12,000*g*, 20 min) and membranes were collected by centrifugation (200,000*g*, 1.5 h). Membranes were resuspended in buffer A and solubilised by incubation with 1% (w/v) n-dodecyl-ß-D-maltoside (DDM; Anatrace) and 0.1 % Cholesteryl hemisuccinate (CHS; Anatrace) for 1 hour at 4°C. Insoluble material was removed by centrifugation (100,000*g*, 30 min) and the supernatant applied to a 5 ml streptactin XT 4flow purification column (iba) and the column washed with 10 column volume (CV) of buffer A supplemented with 0.02 % DDM and 0.002 % CHS. Proteins were eluted in 4 CV of buffer B (50 mM sodium phosphate pH 8.0, 150 mM NaCl and 1 mM EDTA, 0.02 % DDM, 0.002 % CHS and 50 mM biotin). TEV protease (100 μl, 1 mg.ml) was added to the eluate before dialysis into buffer C (20 mM HEPES pH 7.4, 150 mM NaCl, 0.02 % DDM and 0.002 % CHS) using 100,000 molecular weight cutoff (MWCO) SnakeSkin dialysis tubing (ThermoScientific) for 12 hours at 4°C. The sample was concentrated to 0.5 ml using a 100 kDa MWCO Vivaspin 20 (cytiva) centrifugal filter unit and injected onto a Superose 6 Increase 10/300 GL size exclusion column (cytiva) pre-equilibrated in buffer C. Peak fractions were collected and concentrated for Cryo-EM grid making. For PEGylated samples, Methyl-PEG-NHS was added to the sample to a final concentration of 6.5 mM and incubated on ice for 1 hour before Cryo-EM grid making.

### Purification of the SPP-D265A gp160 substrate complex

The gene encoding full-length human SPP including an Asp-265 to Ala mutation and a C-terminal Twin-Strep tag was inserted into the pHR-CMV-TetO_2_ lentiviral transfer vector (Table S2). This vector, alongside packaging and envelope plasmids (psPAX2 and pMD2.G), was transfected into HEK293T Lenti X cells to produce lentivirus. The lentivirus was used to transduce HEK293S GnTI^−^ Tet^R^ cells to generate a stable cell line. This cell line was subsequently transduced with lentivirus generated from a second pHR-CMV-TetO^2^ lentiviral transfer vector carrying the gene encoding residues 1–32 of gp160 (containing sequence encoding the signal peptide) and a C-terminal hexa-His tag. Cells were cultured and proteins expressed in the same was as described above for wild type SPP.

For purification, cell pellets were resuspended in Buffer A (50 mM sodium phosphate pH 8.0, 150 mM NaCl and 1 mM EDTA) supplemented with DNase I (30 μg/ml) and a cOmplete EDTA-free Protease Inhibitor Cocktail tablet (Sigma) for 30 minutes before passing through an EmulsiFlex C5 homogenizer (Avestin) at 15,000 psi. Lysates were clarified by centrifugation (12,000*g*, 20 min) and membranes were collected by centrifugation (200,000*g*, 1.5 h). Membranes were resuspended in buffer A and solubilised by incubation with 1% (w/v) n-dodecyl-ß-D-maltoside (DDM; Anatrace) and 0.1 % Cholesteryl hemisuccinate (CHS; Anatrace) for 1 hour at 4°C. Insoluble material was removed by centrifugation (100,000*g*, 30 min). The supernatant was applied to a 5 ml streptactin XT 4flow purification column (IBA) and the column washed with 10 column volume (CV) of buffer A supplemented with 0.02 % DDM and 0.002 % CHS. Proteins were eluted in 4 CV of buffer B (50 mM sodium phosphate pH 8.0, 150 mM NaCl and 1 mM EDTA, 0.02 % DDM, 0.002 % CHS and 50 mM biotin). The eluate was adjusted to contain 2 mM imidazole and incubated with 1 ml of cOmplete His-tag purification resin (Roche) for 1 hour. The resin was washed with 20 CV of buffer C supplemented with 2 mM imidazole. Proteins were eluted with 6 CV of buffer C containing 200 mM imidazole, concentrated to 0.5 ml and injected onto Superose 6 Increase 10/300 GL size exclusion column (cytiva) pre-equilibrated in buffer C. Peak fractions were collected and concentrated. For PEGylated samples, Methyl-PEG-NHS was added to the sample to a final concentration of 6.5 mM and incubated on ice for 1 hour before Cryo-EM grid making.

### Cryo-EM sample preparation and imaging

Purified wild type SPP (A280_nm_ = 6.0, 4 μl), PEGylated wild type SPP (A280_nm_ = 7.0, 4 μl), PEGylated SPP-D265A - substrate complex (A280_nm_ = 4.7 – 6.7, 4 μl) were adsorbed to glow discharged holey carbon-coated grids (Quantifoil 300 mesh, Au R1.2/1.3 for 15 s at 15 mA). Grids were then blotted for 2 s at 100% humidity at 4°C and frozen in liquid ethane using a Vitrobot Mark IV (FEI). Data were collected in counted mode on a Titan Krios G4 (Thermo Scientific) operating at 300 kV with a Selectris X imaging filter (Thermo Fisher Scientific) with slit width of 10 eV at 165,000x magnification on a Falcon 4 direct detection camera (Thermo Fisher Scientific), with a pixel size was 0.732 Å. Movies were collected at a total dose of 51.1 – 62.9 e^−^/A^2^ (Fig. S10).

### Cryo-EM data processing

Patched motion correction, CTF parameter estimation, particle picking, extraction, and initial 2D classification were performed in SIMPLE 3.0^54,55^. All downstream processing was carried out in cryoSPARC^56^ or RELION^57^, using the csparc2star.py script within UCSF pyem^58^ to convert between formats. Global resolution was estimated from gold-standard Fourier shell correlations (FSCs) using the 0.143 criterion and local resolution estimation was calculated within cryoSPARC using an FSC threshold of 0.5 or within RELION^57^.

The cryo-EM processing workflow for wild type SPP is outlined in Fig. S1. Briefly, particles from dataset 1 were subjected to two rounds of reference-free 2D classification (k=200) within cryoSPARC. Three volumes were then generated from a 2,083,641-particle subset after multi-class *ab initio* reconstruction using a maximum resolution cutoff of 12 Å. Particles (1,055,491) from the most populated class were selected for a second multi-class *ab initio* reconstruction. The three volumes from the first multi class *ab initio* reconstruction and the most populated volume from the second multi-class *ab initio* reconstruction were used as reference volumes for all subsequent heterogenous refinements. Particles from datasets 2 and 3 were subjected to two rounds of heterogenous refinement. Particles (1,700,232) from the most populated and structured classes were combined and non-uniform refined to generate a 4.1 Å volume. These particles were subjected to a heterogenous refinement followed by two rounds of multi class *ab initio* reconstructions. Particles (282,390) were Bayesian polished in RELION. A final non-uniform refinement in cryoSPARC yielded a 3.8 Å volume.

The cryo-EM processing workflow for the SPP - substrate complex is outlined in Fig. S5. Briefly, particles from dataset 1 were subjected to two rounds of reference-free 2D classification (k=200) within cryoSPARC. Six volumes were then generated from a 903,820-particle subset after multi-class *ab initio* reconstruction using a maximum resolution cutoff of 12 Å. These six volumes were used as reference volumes for all subsequent heterogenous refinements. A larger particle subset from the second 2D classification was used as input for a heterogenous refinement and the particles (416,758) from the most populated and structured class retained for use later. Particles from dataset 2 were subjected to two rounds of reference-free 2D classification (k=200) within cryoSPARC. A particle subset (2,601,006) was used as input for a heterogenous refinement and the particles from the most populated and structured class used as input for a multi class *ab initio* reconstruction. Particles (671,838) from two classes were retained for later use. Particles from dataset 3 were subjected to two rounds of reference-free 2D classification (k=200) within cryoSPARC. A particle subset (1,631,064) was subjected to two rounds of multi class *ab initio* reconstruction. Particles (213,057) from the most structured class were retained for use later. Particles from dataset 4 were subjected to two rounds of reference-free 2D classification (k=200) within cryoSPARC. A particle subset (3,110,394) was used as input for a heterogenous refinement and the particles (1,017,094) from the most populated and structured class retained for later use. Particles retained from datasets 1–3 were used as input for a heterogenous refinement and the particles (794,900) from the most populated and structured class combined with the particles retained from dataset 4. These combined particles were non-uniform refined to generate a 4.2 Å volume. Particles (1,811,994) were Bayesian polished in RELION and non-uniform refined in cryoSPARC to generate a 4.1 Å volume. Another heterogenous refinement and non-uniform refinement generated a 4.0 Å volume.

### Cryo-EM model building and refinement

Atomic models were built into their respective cryo-EM volumes in Coot^59^. Models were further refined in real-space using PHENIX^60^ with rotamer, Ramachandran restraints, and secondary structure restraints (where necessary) against the deepEMhancer maps, yielding the model described in Table S1. All models were validated using MolProbity within PHENIX^61^. Figures were prepared using UCSF ChimeraX^62^ and Adobe Illustrator.

### Cell-based cleavage assay

Human embryonic kidney T-REx-293 Flip In cells (Flp-In T-REx-293; Invitrogen) cells and *SPP* knockout (*SPP*-KO) Flp-In T-REx-293 cells were cultured in DMEM (Gibco) supplemented with 10% fetal bovine serum (FBS, Gibco) and 1 % L-glutamine. Wild type and *SPP*-KO Flp-In T-REx-293 cells were transfected in T25 flasks 24 hours before transfection. Plasmid encoding SPP - 3xFLAG (50 ng) or its variants and plasmid encoding gp160 residues 1–32 with a C-terminal Myc tag or plasmid encoding CYP51A1 residues 1–61 with a C-terminal Myc-tag were co-transfected using a 3:1 ratio of Mirus TransIT-LT1 transfection reagent (μl) : DNA (μg) both of which were diluted in OptiMEM (Gibco). 24 hours after transfection, cells were washed once in PBS, detached using TrypLE Express (Gibco), the trypsin reaction quenched with FBS, cells washed with PBS before lysis in 2x SDS sample buffer (0.25 M Tris-HCl pH6.8, 10% SDS, 50% glycerol, 0.02% bromophenol blue) supplemented with 100 mM DTT and 1% Triton X100. Proteins were separated by SDS–PAGE and detected by immunoblotting using antibodies against FLAG-tag (SPP), Myc-tag (gp160) or GAPDH.

### Affinity chromatography co-purification assays

Human embryonic kidney 293F (HEK293F; ThermoFisher) cells were cultured in FreeStyle 293 Expression medium (Gibco) supplemented with 1% non-essential amino acids and 1% foetal bovine serum (FBS, Gibco). Suspension cells were grown at 37 °C, 130 rpm, and 8% CO_2_ to a cell density of 2 × 10^6^ cells per ml. Plasmid encoding SPP - Twin-Strep (6 μg) or its variants and plasmid encoding gp160 residues 1–32 with a C-terminal 3xMyc tag (12.5 μg) were co-transfected using a 3:1 ratio of Polyethylenimine Hydrochloride (PEI, MW 40,000, μl) : DNA (μg) into 25 ml of HEK293F cells. Cells were harvested two days post induction by centrifugation (1500 rpm, 10 min, 4°C). Cells were resuspended in 1 ml of buffer A supplemented with DNase I (30 μg/ml), cOmplete EDTA-free Protease Inhibitor Cocktail tablet (Sigma), 1% DMM w/v and 0.1% CHS (w/v) before incubating for 1 hour at 4°C. Samples were clarified by centrifugation (30,000*g*, 20 min) and incubated with 100 μl Streptactin XT 4Flow resin (IBA) for 1 hour. The resin was washed with buffer A supplemented with 0.02 % DDM and 0.002 % CHS and proteins eluted from the resin with buffer B. Samples were resuspended in 2x SDS sample buffer (0.25 M Tris-HCl pH6.8, 10% SDS, 50% glycerol, 0.02% bromophenol blue) supplemented with 100 mM DTT. Proteins were separated by SDS–PAGE and detected by immunoblotting using antibody against Myc-tag (gp160) or detected using Strep-Tactin-HRP conjugate against Strep-tag (SPP).

### Co-immunoprecipitation

The day before the experiment, approximately 5×10^6 cells were seeded in a 10 cm dish format, and protein expression was induced with doxycycline for 24 hours. The next day, cells in 10cm dishes were washed with cold PBS on ice and lysed by scraping in 1% DMNG (Anatrace) lysis buffer (50mM Tris-HCl pH 7.5, 150mM NaCl) containing cOmplete protease inhibitor cocktail (Roche). The lysates were transferred into ice cold 2mL Eppendorf tubes and rotated head-over-head for 1 hour at 4oC. Cell nuclei and debris were pelleted by centrifugation in a pre-cooled centrifuge at 20000xg for 20 minutes. Post-nuclear supernatants were isolated and incubated with anti-FLAG magnetic beads (Pierce^™^, Thermo Fischer Scientific) in the cold room with head-over-head rotation for 2 hours. After three washes in 0.1% DMNG washing buffer (50mM Tris-HCl pH 7.5, 150mM NaCl), proteins were eluted in 1x sample buffer for 20 min at 37°C or using 3xFLAG peptide (500 ng/uL; Sigma-Aldrich) for 30 min on ice. The eluate was transferred to a new 1.5mL Eppendorf tube and denatured by adding Laemmli sample buffer containing DTT. Denatured material was either stored in −20°C or used directly for immunoblotting.

### Flow cytometry

The model substrate consisted of the first 61 amino CYP51A1, encompassing an N-terminal AMPH followed by a single TM domain and is expressed as a fusion to sfGFP and 3×HA tags. Analysis was performed 16 h after the time of doxycycline induction in cells. Cells were trypsinized, washed once with ice-cold PBS and re-suspended in FACS buffer (1 mM EDTA, 2% FBS in PBS). Cells were then assessed for expression of target constructs by fluorescence using a BD LSRFortessa X-20, and data were processed using FlowJo 10.4 (https://www.flowjo.com/). At least 10,000 cells per sample were analysed, gating on the main population in the forward scatter/side scatter (FSC/SSC) plot.

### Modeling of unresolved regions and molecular dynamics (MD) simulations

Atomic coordinates from the cryo-EM structures were used for residues 24–55, 79–223 and 240–340 of the *apo* dimer, and residue 30–55 and 67–349 of the substrate-bound dimer. Initial coordinates for the unresolved loop regions were modeled through AlphaFold 3^63^, using each cryo-EM structure as a template in the prediction of the respective state, as well as disabling the use of multiple-sequence alignments. Unresolved terminal regions (residues 1–23 and 341–377 for the apo state, 1–29 and 350–377 for the substrate-bound state) were also excluded from the query sequence. Because the use of a template does not enforce an exact match between predicted coordinates and experimentally-determined coordinates, nor does it inform the predicted supra-molecular arrangement of the complex, a set of 20,000 models was generated for each state, and the best-fit model for each was selected based on the lowest C-alpha RMSD from the experimental coordinates (1.25 Å for the apo state, 1.50 Å for the substrate-bound). Finally, experimentally-resolved coordinates were placed in each structure, while the predicted coordinates were energy-minimized with the all-atom CHARMM force field^64^, with electrically-neutral caps at the ends of each peptide chain, and the Asp219 residue of each SPP chain in protonated form.

Coarse-grained (CG) simulation models were generated for each state using the MARTINI 3 force field^65^. Owing to the known limitations of CG force fields in preserving tertiary and quaternary protein structures, the experimental structure of each dimer was preserved by adding harmonic bonds of a force constant of 10 kJ/mol/Å^2^ between all CG particles within a distance of 15 Å. This cutoff distance was chosen to allow for at least five pairs of particles from the opposing protomers to be linked by a bond, in addition to those in the β-sheet connecting the protomers at the cytosolic side of the structure. Each SPP dimer structure was embedded in a 300×300 Å^2^ bilayer of POPC molecules using the Insane tool^66^, with a ~70 Å water buffer between the lipid bilayer and its periodic images. The same procedure was repeated for the first protomer of each structure, using the dimer structure as a basis. Molecular dynamics (MD) simulations were carried out with GROMACS version 2025^67^, with stochastic rescaling of velocities and periodic cell vectors^68,69^ to maintain a 300 K temperature, 1 atm isotropic pressure and zero membrane tension through semi-anisotropic pressure coupling. Collective-variable restraints were applied through the Colvars library^70,71^ to the relative position of the dimer in the bilayer plane (“distanceXY” variable), as well as the angle of rotation around the membrane-normal axis (“spinAngle” variable), thus enabling membrane deformations to be sampled across the entire bilayer plane. For each system, six independent replicas were simulated, each for 20 μs (dimer simulations) or 5 μs (protomer simulations). Trajectories were considered for analysis only if both lipid bilayer and water solution remained in fluid phase throughout.

To analyze the membrane properties of each bilayer, the average coordinates of the phosphate groups of the lipid molecules were projected onto a 2-dimensional grid with grid-cell dimensions of 2.5×2.5 Å^2^. Curvature radius *R* was estimated by fitting the midplane’s positions with a sinusoidal function extending over the bilayer inside of a 50 Å-wide band; errors are standard deviations over independent replicas.

## Supplementary Material

Supplementary Files

This is a list of supplementary files associated with this preprint. Click to download.

- SupplementalData.docx

## Data Availability

Cryo-EM reconstructions and atomic models for human SPP (EMD-75261; PDB 10LC) and human SPP-substrate complex (EMD-75262; PDB 10LF) have been deposited in the Electron Microscopy Data Bank and Protein Data Bank, respectively, with the appropriate accession codes listed in parenthesis beside each entry.
